# Integrated genomic and transcriptomic analysis revealed mutation patterns of de-differentiated liposarcoma and leiomyosarcoma

**DOI:** 10.1186/s12885-020-07456-2

**Published:** 2020-10-28

**Authors:** Wenshuai Liu, Hanxing Tong, Chenlu Zhang, Rongyuan Zhuang, He Guo, Chentao Lv, Hua Yang, Qiaowei Lin, Xi Guo, Zhiming Wang, Yan Wang, Feng Shen, Shengzhou Wang, Chun Dai, Guan Wang, Jun Liu, Weiqi Lu, Yong Zhang, Yuhong Zhou

**Affiliations:** 1 Department of General Surgery, Shanghai Public Health Clinical Center, Fudan University, Shanghai, China; 2 Department of General Surgery, Zhongshan Hospital, Fudan University, Shanghai, China; 3 Department of Medical Oncology, Zhongshan Hospital, Fudan University, Shanghai, China; 4 GenomiCare Biotechnology (Shanghai) Co. Ltd., Shanghai, China

**Keywords:** Soft tissue sarcoma, Gene fusion, Tumor microenvironment, Immune infiltration

## Abstract

**Background:**

Treating patients with advanced sarcomas is challenging due to great histologic diversity among its subtypes. Leiomyosarcoma (LMS) and de-differentiated liposarcoma (DDLPS) are two common and aggressive subtypes of soft tissue sarcoma (STS). They differ significantly in histology and clinical behaviors. However, the molecular driving force behind the difference is unclear.

**Methods:**

We collected 20 LMS and 12 DDLPS samples and performed whole exome sequencing (WES) to obtain their somatic mutation profiles. We also performed RNA-Seq to analyze the transcriptomes of 8 each of the LMS and DDLPS samples and obtained information about differential gene expression, pathway enrichment, immune cell infiltration in tumor microenvironment, and chromosomal rearrangement including gene fusions. Selected gene fusion events from the RNA-seq prediction were checked by RT-PCR in tandem with Sanger sequencing.

**Results:**

We detected loss of function mutation and deletion of tumor suppressors mostly in LMS, and oncogene amplification mostly in DDLPS. A focal amplification affecting chromosome 12q13–15 region which encodes *MDM2*, *CDK4* and *HMGA2* is notable in DDLPS. Mutations in *TP53*, *ATRX*, *PTEN*, and *RB1* are identified in LMS but not DDLPS, while mutation of HERC2 is only identified in DDLPS but not LMS. RNA-seq revealed overexpression of *MDM2*, *CDK4* and *HMGA2* in DDLPS and down-regulation of *TP53* and *RB1* in LMS. It also detected more fusion events in DDLPS than LMS (4.5 vs. 1, *p* = 0.0195), and the ones involving chromosome 12 in DDLPS stand out. RT-PCR and Sanger sequencing verified the majority of the fusion events in DDLPS but only one event in LMS selected to be tested. The tumor microenvironmental signatures are highly correlated with histologic types. DDLPS has more endothelial cells and fibroblasts content than LMS.

**Conclusions:**

Our analysis revealed different recurrent genetic variations in LMS and DDLPS including simultaneous upregulation of gene expression and gene copy number amplification of *MDM2* and *CDK4*. Up-regulation of tumor related genes is favored in DDLPS, while loss of suppressor function is favored in LMS. DDLPS harbors more frequent fusion events which can generate neoepitopes and potentially targeted by personalized immune treatment.

## Background

Soft tissue sarcoma (STS) is a rare malignant tumor but has a great diversity. It occurs about 2.91 times per 100,000 people and accounts for 1.05% of overall cancer incidents in China [1]. However, there are more that 50 recognized STS subtypes and that makes the accurate diagnosis and treatment a challenge [2]. LPS and LMS are among the most frequent STS subtypes, accounting for 5.75 and 5.97% of all cases respectively [1]. At the same time, LMS and a subtype of LPS, DDLPS, are the two most aggressive types, both having a complex karyotype and high recurrence rate [3]. Nevertheless, they differ significantly in histology, biology, and clinical behaviors [4]. For example, DDLPS tends to recur locally, while LMS has a higher chance of distant recurrence. It is unclear what genetic and molecular differences may have contributed to the distinction between LMS and DDLPS. In order to gain insight about their origins and molecular difference, and enlighten differential treatment, we performed integral WES and RNA-seq analysis and compared the two subtypes. We identified potential key mutational events that may have differentially driven tumorigenesis and discovered different tumor microenvironments in LMS and DDLPS. We also detected few neoantigens, mostly in DDLPS, which may be targeted by personalized immune therapy.

## Methods

### Patients and specimens

Twenty LMS and twelve DDLPS tissue samples and matched peripheral blood from the same patient were collected at Zhongshan Hospital of Fudan University with patient consent. The study protocol was reviewed and approved by the Ethics Committee of Zhongshan Hospital. H&E stained histological sections were reviewed by an expert pathologist to confirm subtype diagnosis and tumor content to be above 20%. The cytogenetic, immunohistochemistry (IHC), and pathologic lab data, as well as clinical treatment and outcome records were manually curated from the electronic medical records of the hospital.

### WES

DNA in formalin-fixed, paraffin-embedded (FFPE) tissue samples was extracted using MagMAX FFPE DNA/RNA Ultra kit (cat# A31881, ThermoFisher). DNA in snap-frozen tissue and peripheral whole blood was extracted using Maxwell RSC blood DNA kit (cat# AS1400, Promega). Purified DNA was sheared with a Covaris L220 sonicator and hybridized to the probes in Agilent SureSelect XT Human All Exon V7 kit (cat# 5991–9039, Agilent) for exome enrichment. Captured exome DNA was PCR amplified, end-repaired, and attached to the adapters and barcode using SureSelect XT HS and Low Input Library Preparation Kit for ILM (Pre PCR) kit (cat# G9704, Agilent, Santa Clara, CA, USA) according to the manufacturer’s specification. The prepared libraries were sequenced on an Illumina NovaSeq-6000 Sequencing System to generate 150 × 150-bp paired-end reads. The image analysis and base calling were done using the Illumina onboard RTA3 program with default parameters. After removing adapters and low-quality reads, the reads were aligned to NCBI human genome reference assembly hg19 using the Burrows-Wheeler Aligner (BWA) alignment algorithm and further processed using the Genome Analysis Toolkit (GATK, version 3.5), including the GATK Realigner Target Creator to identify regions that needed to be realigned. Single-nucleotide variants (SNV), Indel, and copy number variation (CNV) were determined using the MuTect/ANNOVAR/dbNSFP31, VarscanIndel, and CNVnator softwares respectively as reported in [5]. During the mutation calling, the reads from the tumor sample were compared with the paired blood from the same patient to generate a list of somatic mutations. The called somatic mutations were then filtered and annotated using Variant Effect Predictor (VEP) package (hg19 version) [6].

Mutation analysis and visualization were performed using the R (v3.3.3) (http://www.r-project.org), Bioconductor (v3.4) (http://www.Bioconductor.org), and MAFtools softwares [7, 8]. Mutation data in maf format was generated using VCF2MAF v1.6.16 [9]. CNA of individual samples was determined using WES data by Control-FREEC software [10], with the window size set to 500 bp and step size set to 250 bp as recommended by the software authors. Recurrent focal and broad CNA in patient group were identified by GISTIC2.0 and GSEA [11, 12].

### Tumor mutational burden and microsatellite status

Absolute counts of the tumor mutational burden (TMB) were calculated from the total number of nonsynonymous somatic mutations detected in WES using a published algorithm [13]. Autosomal microsatellite trains containing 1–5 bp repeating subunits in length and comprising five or more repeats referenced to GRCh37/hg19 were identified using MISA (http://pgrc.ipk-gatersleben.de/misa/misa.html) [14]. Microsatellite Instability (MSI) score was defined as “number of unstable microsatellite sites / total valid sites” and a score ≥ 3.5% is regarded as high (MSI-High) and < 3.5% as low (MSI-Low) [15].

### RNA-Seq

RNA was purified from FFPE tissue using the MagMAX FFPE DNA/RNA Ultra kit, reverse-transcribed using the NEBNext RNA First Strand Synthesis Module (cat# E7525S, NEB) and NEBNext Ultra II non-directional RNA Second Strand Synthesis Module (cat# E6111S, NEB) for cDNA Synthesis. RNA-seq libraries were prepared from the cDNA using SureSelect XT HS and Low Input Library Preparation Kit for ILM (Pre PCR) (cat# G9704, Agilent, Santa Clara, CA, USA) following the manufacturer’s instruction. The libraries were sequenced on an Illumina NovaSeq-6000 Sequencing System to generate 150 × 150 paired-end reads. Using FFPE sample for RNA-Seq is a more difficult task than using fresh or snap-frozen tissue. However, FFPE is still the most accessible tumor samples so studies have been lunched to test the use of RNA from FFPE sample for RNA-seq analysis and established reliable protocols [16, 17]. The protocols have since been adopted to routine use in oncogenomics studies and commercial kits have been developed by several suppliers, including the two New England Biolabs kits (NEBNext RNA first strand synthesis module and NEBNext Ultra II non-directional RNA second strand synthesis module) we used above.

### Differential gene expression analysis

Reads from RNA-Seq were quality checked, filtered, and aligned to reference genome (hg19) using STAR (version 020201) and assembled using StringTie2 (version 1.3.5) [18, 19]. A count matrix of raw reads was generated and the reads were further normalized by the upper quartile counts. A list of genes expressed differentially between LMS and DDLPS was compiled using DESeq2 [20]. The significance threshold was set at adjusted *P*-value < 0.05 and Log2 fold change (LogFC) > 1.

### Pathway analysis

Biological pathway analysis was done with the Kyoto Encyclopedia of Genes and Genomes (KEGG) pathway analysis and Gene Set Enrichment Analysis (GSEA) softwares using the above gene expression data and visualized by the NetworkAnalyst (http://www.networkanalyst.ca) software [12, 21, 22].

### Gene fusion analysis

#### NGS sequencing and bioinformatic prediction

STAR FUSION 1.8.0 was used to identify gene fusion events from the above mapped RNA-seq reads [23]. The standard setting, except a custom filter of at least 3 positive reads per event, was applied to increase calling stringency.

#### Reverse transcription PCR (RT-PCR) and sanger sequencing confirmation

Extracted RNA from tumor tissue was reverse transcribed using SuperScript Double Stranded cDNA synthesis kit (cat#11917010, Invitrogen) and random hexamer oligos as the primer. The produced cDNA was amplified by PCR using a left primer complementary to the upper stream sequence and a right primer complementary to the downstream sequence across the fusion junction as predicted by the above NGS-STAR FUSION pipeline. The primer sequences, fusion junction sequences and reads number are listed in Supplementary Table S1. The PCR product was isolated by electrophoresis on agarose gel, extracted, and cloned in pCR4-TOPO vector (cat#450030, ThermoFisher), then subjected to Sanger sequencing to verify the fusion event.

### Immune classification of STS

The Microenvironment Cell Populations-counter (MCP-counter) method was used to profile tumor microenvironment infiltrating immune cells with RNA-seq derived Transcripts Per Million (TPM) matrix generated by the stringtie2 pipeline [24, 25]. Abundance scores for eight immune populations (T cells, CD8+ T cells, cytotoxic lymphocytes, natural killer cells, B cell lineage, monocytic lineage, myeloid dendritic cells and neutrophils), and two stromal populations (endothelial cells and fibroblasts) are calculated using the signature composition defined previously [24]. Unsupervised clustering of samples was performed based on the metagene Z-score of MCP-counter.

### Statistical analysis

Statistical analysis was performed with R (version 3.6.3) or SPSS (version 25). Unless stated otherwise, unpaired t-test was used in all group comparisons. A result of *p* < 0.05 was considered significant.

## Results

Genomic analysis has greatly expanded people’s knowledge in the genetic composition of cancer mutations and in many cases helped design of new treatments. In the hope of using genomic information to guide the diagnosis and treatment of STS, we recruited thirty-two patients, twenty with LMS and twelve with DDLPS, at Zhongshan Hospital of Fudan University, Shanghai, China. The patient characteristics are indicated in Table 1.

**Table 1:** Summary of Clinical information and WES and RNA-seq analysis

	All	LMS	DDLPS
Sample size			
WES	32	20	12
RNA-seq	16	8	8
Age (years)			
Median	51	49.5	54
Range	16–70	16–65	37–70
Gender (%)			
Male	12 (37.5%)	3 (15%)	9 (75%)
Female	20 (62.5%)	17 (85%)	3 (25%)
WES			
TMB (counts/Mb)			
Median	2.27	2.61	1.97
Range	1.20–16.1	1.57–16.1	1.20–4.51
MSI score (%)			
High	1 (3.1%)	1 (5%)	0 (0%)
Low	31 (96.9%)	19 (95%)	12 (100%)
SNP + Indel	2887	2110	1088
CNA gain		113	1089
RNA-seq			
Up-regulated		2404	2364
Gene fusion	40	4	36
RT-PCR verified	8/12 (66.7%)	1/4 (25%)	7/8 (87.5%)

* TMB is reported as counts/Mb. It is calculated by all nonsynonymous mutations detected in WES divided by 35 Mb. 35 Mb is the sum of exome probes in WES

*WES* whole exome sequencing, *TMB* tumor mutation burden, *MSI* microsatellite instability

### Somatic mutation profile

**Fig. 1:**
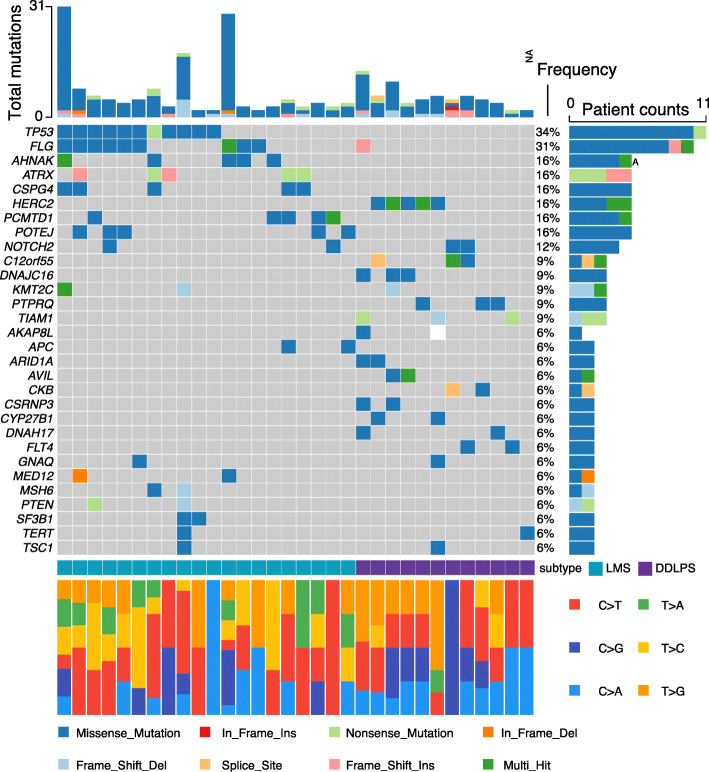
Top30 frequently mutated genes in LMS and DDLPS. Patients are grouped by disease (Cerulean: LMS; Purple: DDLPS) and the genes are ranked by their mutation frequencies. The top chart indicates total number of mutations in each patient. The types of mutation and nucleotide transition are color coded

Whole exome sequencing was run on both the tumor and paired peripheral blood (normal) samples from the same patient and compared to give somatic mutations in the tumor for each patient. Overall, the tumor mutational burden (TMB) of STS with a median of 2.27 counts/Mb is relatively low in comparison with other cancers [4]. There is no significant difference of TMB between LMS and DDLPS (median 2.61 and 1.97 counts/Mb respectively, *p* = 0.105) (Table 1). A large number of somatic mutations led by *MUC16* (66%) and *PABPC3* (56%) were detected (Supplementary Table S2). In order to focus on the genes with a known function in cancer or reported to related to cancer, we filtered the mutations against a list of 1190 member cancer related genes compiled from literature and OncoKB (http:\crwww.oncokb.org) [26]. There is a distinct distribution of somatic mutations between LMS and DDLPS among the top 30 most mutated cancer-related genes (Fig. 1). Eleven out of the 20 (55%) LMS patients had mutation in TP53. In contrary, none of the DDLPS patient showed TP53 mutation. Similarly, FLG is mutated in 9 (45%) LMS patients but only in 1 (8.3%) DDLPS patient. *ANHAK*, *ATRX*, *CSPG4* and *PCMTD1* each was mutated in 5 (25%) LMS patients and none DDLPS patients. *HERC2* was mutated in 5/8 (41.7%) DDLPS patients, *C12orf55*, *DNAJC16*, *PTPRQ*, and *TIAM1* each was mutated in 3 (25%) DDLPS patients. None of the above five genes had mutation in LMS patients (Fig. 1). As a comparison, the mutation frequency of *HERC2* in TCGA-SARC dataset was 11/58 (19.0%) for DDLPS and 18/102 (17.6%) for LMS cases. The mutation frequency of *TP53* in TCGA-SARC dataset was 10/58 (17.2%) for DDLPS and 80/102 (78.4%) for LMS cases. Therefore *HERC2* mutation is imbalanced and *TP53* mutation is generally lower in both DDLPS and LMS groups in our patients as comparing to that of TCGA-SARC dataset [4]. This may arise from the difference of patient composition. All 32 cases in our group are ethnic Chinese but only 2/160 (1.25%) are Asian and 140/160 (87.5%) are white in the LMS and DDLPS cases of TCGA-SARC dataset. The number of cases is small in both groups though and that prevented us to draw a definitive answer about the distribution difference. The absence of *HERC2* mutation and biased presence of *TP53* mutation in the LMS group is an interesting observation. HERC2 belongs to E3 ubiquitin protein ligases, and can modulate p53 activity through regulating p53 oligomerization independent of MDM2 [27]. Probably because the mutations that affect p53 tetramerization disrupt the HERC2-p53 interaction, therefore *HERC2* mutations are redundant in LMS with mutant *TP53*.

### SCNA in LMS and DDLPS

**Fig. 2:**
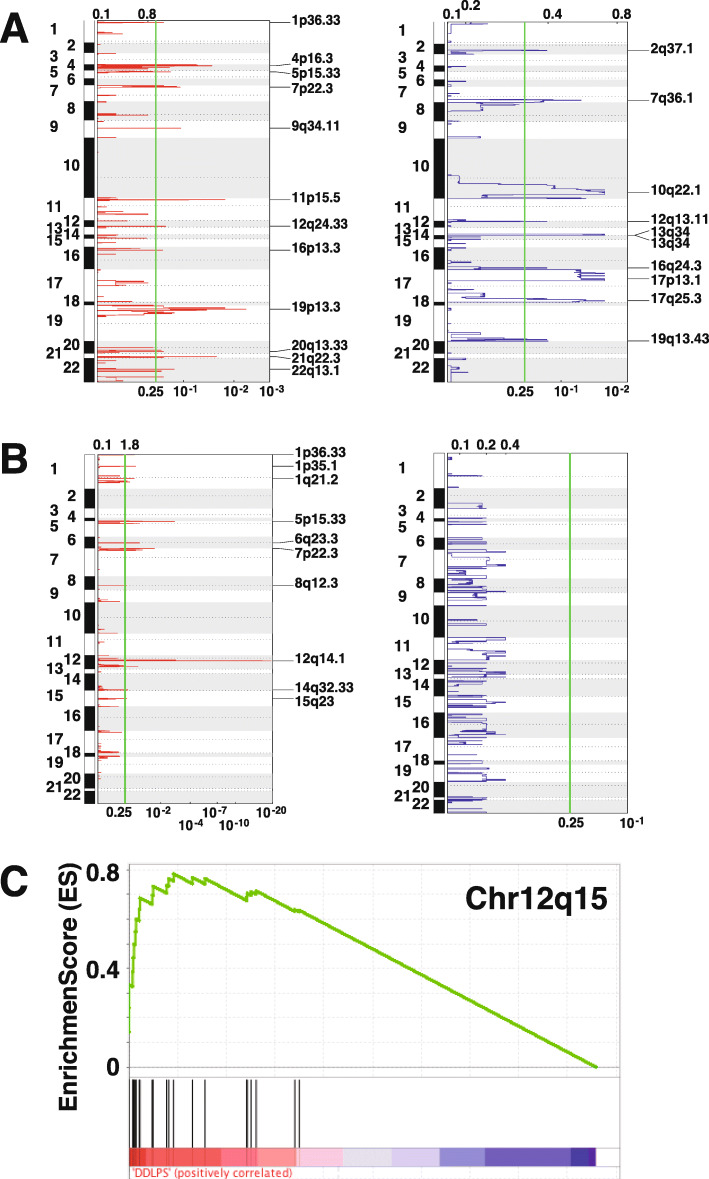
Local gene copy number alteration.**a-b** GISTIC analysis of recurrent amplification (left, red) and deletion (right, blue) in LMS (**a**) and DDLPS (**b**). The y-axes represent genomic position of altered regions (left axis: chromosome; right axis: cytoband) and the x-axes represent normalized amplification signal (top axis) and significance by Q value (bottom axis). The vertical green line represents the significance cutoff at Q value = 0.25. The most prominent amplification is seen around 12q14 in DDLPS. **c** GSEA by position. Gene sets of chromosomal neighbors are used as the input in GSEA which gives an ES = 0.784 (nominal *p*-value 0.0177, FDR q-value 0.447) at Chr12q15. *MDM2* is located at the junction of Chr12q14 and Chr12q15 and on the side of Chrq15

In addition to SNV and indel, gene amplification and deletion are also important contributors to carcinogenesis. To further reveal the somatic copy number alterations (SCNA) in Chinese sarcoma patients, we used GISTIC2.0 to detect SCNA (Supplementary Tables S3–5). We found significant chromosomal loss in LMS peaked at cytobands 10q22.1, 13q34, and 17p13.1, where *PTEN*, *RB1* and *TP53* are located respectively (Fig. 2a). All these genes are important tumor suppressors. This suggested that chromosomal loss affecting tumor suppressor genes is a hallmark of LMS. On the other hand, we found a focal amplification peaked at 12q14.1, included in its wide peak are *MDM2* (12q15), *CDK4* (12q14.1) and *HMGA2* (12q14.3) (Fig. 2b). We further verified the prediction of GISTIC by GSEA analysis under the positional mode. We used chromosomal neighbors as the input gene set instead of signaling pathway members in GSEA and found 12q14-12q15 regions are indeed enriched in DDLPS (EnrichmentScore ES = 0.662 for 12q14 and ES = 0.784 for 12q15) (Fig. 2c and data not shown). *MDM2* and *CDK4* are both important cell cycle regulating genes. HMGA2 is a chromosomal structure organization protein and may also play a role as a transcription factor. It has been reported that alteration of HMGA2 is associated with myxoid liposarcoma and takes part in adipogenesis and mesenchymal differentiation [28, 29]. Amplification of 12q14 and 12q15 regions likely upregulated the above genes important in regulation of cell cycle and transformation of adipocytic tissue, therefore drove DDLPS. At the gene level, we detected co-amplification of *MDM2* and *CDK4* in more than 90% DDLPS cases. Such co-amplification combined with *TP53* inactivation would result in cell proliferation, and is very likely the initiating events to drive fat tumorigenesis in DDLPS [30].

Additional genes detected by GISTIC 2.0 with amplification and deletion are listed in supplement Table S2-S5. In LMS, 96 genes are significantly amplified, while as many as 4532 genes are significantly deleted (confidence level 0.95), indicating that deletion is much more frequent than amplification. In DDLPS 1089 genes are amplified and no genes are significantly deleted. These observations are consistent with previous publications as reviewed in [3]. In DDLPS amplification of oncogenes such as *MDM2*, *CDK4*, *HMGA2* and *JUN *are common, while in LMS deletion of tumor suppressor such as *TP53*, *RB1*, and *PTEN* are preferred, indicating different patterns of CNA in DDLPS and LMS.

### Concurrent and exclusive mutations

**Fig. 3:**
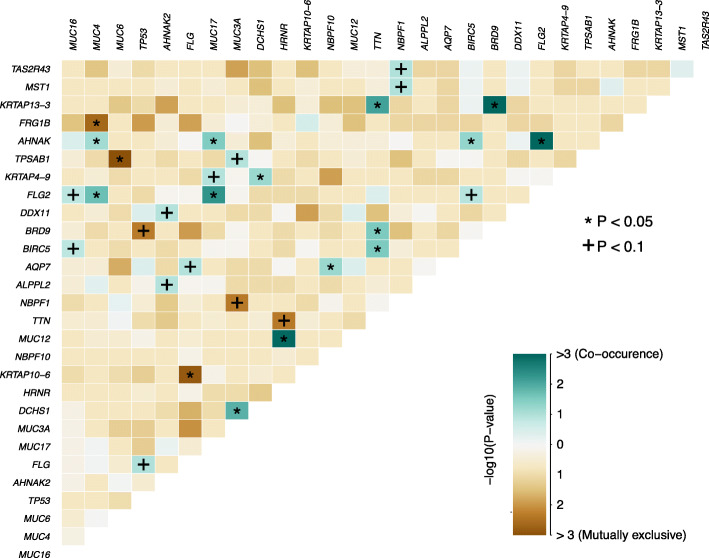
Heatmap of predicted gene interactions in the 32 STS. The color scale represents -log10 *p*-value. The cadet blue indicates co-occurrence and the copper indicates mutual exclusion. Significant *p*-values are indicated by symbols. *: *p <* 0.05; **+**: *p <* 0.1

Given the possibility that synergistic or anti-synergistic interactions between genes may contribute to the origin or progression of LMS and DDLPS, we tested interactions among the somatic mutations called in WES analysis using the somaticInteractions function in MAFtools. (Fig. 3, and the full list in Supplementary Table S2. *TP53* is mutually exclusive with *BRD9* (*p* < 0.1) but co-occurs with Filaggrin (*FLG*) (*p <* 0.1). BRD9 is a subunit of the human BAF (SWI/SNF) nucleosome remodeling complex and has emerged as an attractive therapeutic target in cancer [31]. It has a bromodomain highly homologous to the bromodomain of BRD7, which is reported to interact with p53 and required for p53 function [32]. Whether mutation of *BRD9* promotes LMS through impairment of the p53 pathway is to be determined. *FLG* is a highly mutated cancer driver gene. Its mutation is also found in several other cancer types such as non-melanoma skin cancer, head and neck cancer, lung cancer, colorectal cancer, uterine cancer, and prostate cancer [33]. There has no reported synergic interaction between *FLG* and *TP53* mutations in cancer yet, including STS. The above somatic interaction analysis may provide hints for exploring genes with unspecified functions in STS.

### RNA-seq revealed genes with differential expression

Eight each of the LMS and DDLPS samples that had sufficient RNA quality were further subjected to RNA-Seq analysis. Although fresh or snap-frozen tissue are generally preferred over FFPE samples for RNA extraction and sequencing analysis, FFPE samples are by far the most accessible tissue samples. Dedicated works have been done both by academic labs and commercial manufacturers to develop special protocols to perform RNA-seq from FFPE samples. They have succeeded in using exome capturing for partially degraded RNA. We used a New England Biolabs protocol and companion kits in sample preparation for RNA-seq (see Methods) which has been proven functional and are widely used in NGS work with clinical samples [16, 17].

**Fig. 4:**
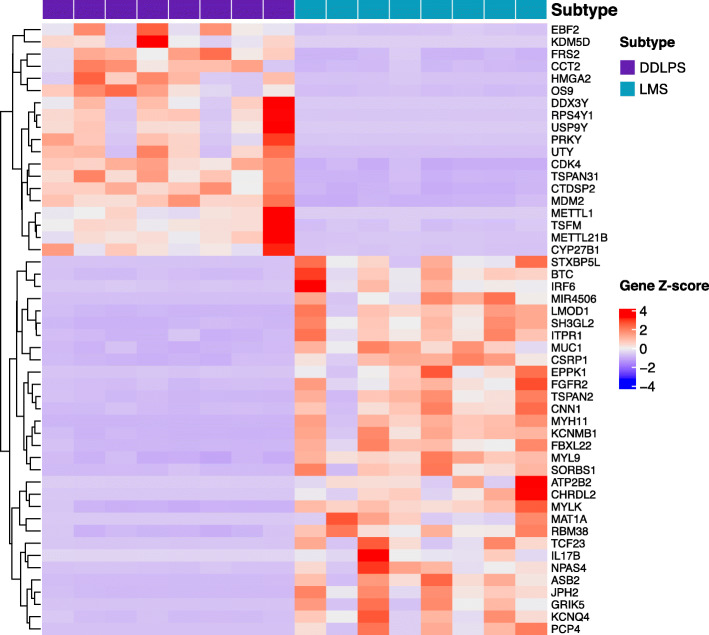
Heatmap of top 50 differentially expressed genes between LMS (cerulean) and DDLPS (purple) with unsupervised clustering. One column represents one sample. The heat scale represents Z-score normalized gene expression. The red indicates up-regulated and the blue indicates down-regulated genes in DDLPS compared with LMS

**Fig. 5:**
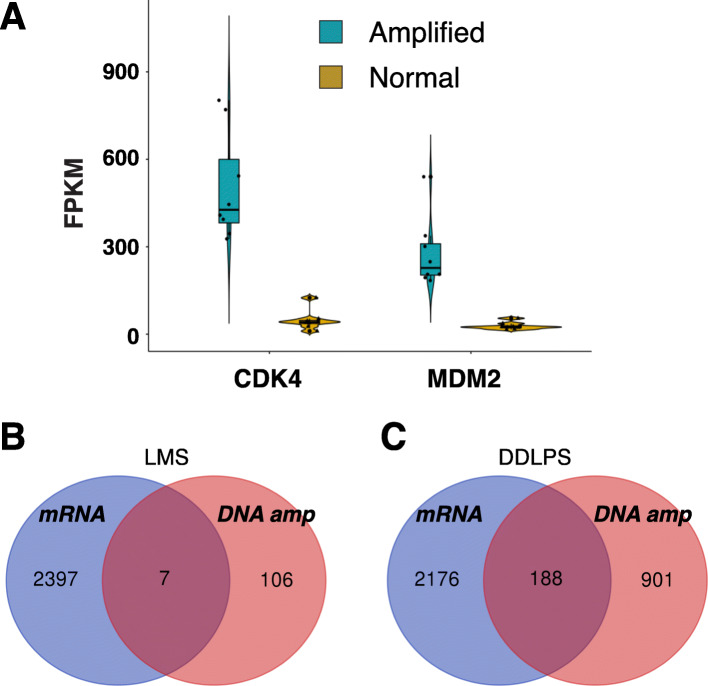
Correlation of gene amplification detected in WES with transcripts upregulation detected in RNA-seq. **a** Violin-box plot comparing the expression of *CDK4* (left) and *MDM2* (right) in patients with (cerulean) and without (gold) gene amplification. The y-axis represents normalized reads number in FPKM detected in RNA-seq. The median value is indicated by the line inside the box. The width of the color shade indicates the distribution frequency. **b-c** Venn diagrams of detected genes amplified in WES (pink) and upregulated in expression in RNA-seq (blue) in LMS (**b**) and DDLPS (**c**) respectively

In total we identified 2396 genes expressed with significantly different levels in the LMS and DDLPS (Fig. 4 and Supplementary Table S6). Unsupervised clustering was performed to examine the discriminant effect of these genes. We found that all the LMS naturally clustered together, and so did the DDLPS samples. Ranked the highest in differential expression is *MDM2*, with logFC = 4.12 (DDLPS over LMS) and adjusted *P*-value = 1.73e-52, consistent with previous studies [4, 34]. *MDM2* is a proto-oncogene. It encodes a nuclear-localized E3 ubiquitin ligase and plays an important role in cell cycle regulation. Its copy number gain has been implicated in cancers including DDLPS [35, 36]. We also observed the gene copy number of *MDM2* is correlated with its mRNA in DDLPS (Fig. 5a). Similarly, another key cell cycle regulator *CDK4* is both highly upregulated in expression (logFC = 3.61, adjusted *p*-value = 1.09e-20) and amplified in gene copy number (Fig. 5a). These results suggest that hyper activation of cell cycle is highly correlated with and may possibly a driving force underlying DDLPS. This notion is supported by a recent study in human mesenchymal stem cell model in which co-expression of *MDM2* and *CDK4* produced DDLPS-like morphology [37]. Additionally, we found *JUN* upregulation (logFC = 1.45, adjusted *P*-value = 0.0396) in DDLPS (Supplementary Table S6). *JUN *is a known player in blocking adipocytic differentiation so may have assisted tumorigenesis in adipose tissue.

Since we detected both CNA in WES and differential gene expression in RNA-seq, it would be of interest to see whether CNA is correlated with gene expression. Although the top varied genes such as *MDM2* and *CDK2* indeed have a good correlation between their DNA and mRNA level (Fig. 5a), the bulk of genes do not show this relationship, especially in the case of LMS (Fig. 5b-c). This may due to several factors. Gene expression is a tightly regulated cellular process. The copy number of available template DNA, especially for those which only have a moderate increase as the bulk of genes are, is only a small contributor of gene expression activity. The more important regulation can be attributed to the accessibility of the chromosomal region, and activities of promoters, specific transcription factors, and DNA dependent RNA polymerase machinery, and mRNA stability.

### Pathway analysis of genes with differential expression

To investigate the pathways affected in LMS and DDLPS, we performed Gene Set Enrichment Analysis (GSEA) (Table 2 and Supplementary Table S7) [12, 21]. We found several pathways are enriched in DDLPS (ES > 0) while depleted in LMS (ES < 0). The pathways enriched in DDLPS include “Ubiquitin mediated proteolysis” (ES = 0.472, adjusted *p*-value = 0.002) which has *MDM2* as a member. The pathways apparently enriched in LMS such as “Calcium signaling pathway”, “Vascular smooth muscle contraction”, and “Linoleic acid metabolism”(ES = − 0.37, − 0.44, and − 0.60 respectively, and adjusted *P*-values = 0.002 for all) are probably due to the difference of the tissue origins rather than tumorigenesis [38, 39]. Another notable pathway is spliceosome (ES =0.53, adjusted *p-*value =0.003) because alteration in transcript splicing may result in different sets of antigens differentially recognizable by immune cells in different tumor environments.

**Table 2:** Pathways enriched in GSEA (LMS in relative to DDLPS)

Name	Total	Hits	EnrichmentScore	*p*-val	p-adj
Calcium signaling pathway	68	58	−0.37	0.00017	0.0028
Vascular smooth muscle contraction	30	22	−0.44	0.000017	0.0028
Linoleic acid metabolism	30	26	−0.60	0.00018	0.0028
Ascorbate and aldarate metabolism	34	28	−0.67	0.00018	0.0028
Chronic myeloid leukemia	33	27	0.53	0.000022	0.0028
p53 signaling pathway	31	27	0.50	0.000022	0.0028
Spliceosome	27	22	0.52	0.000022	0.0028
AGE-RAGE signaling pathway in diabetic complications	18	16	0.47	0.000023	0.0028
Ubiquitin mediated proteolysis	27	25	0.47	0.000023	0.0028
Osteoclast differentiation	44	39	0.50	0.000023	0.0028

### Distinct gene fusion patterns between DDLPS and LMS

**Fig. 6:**
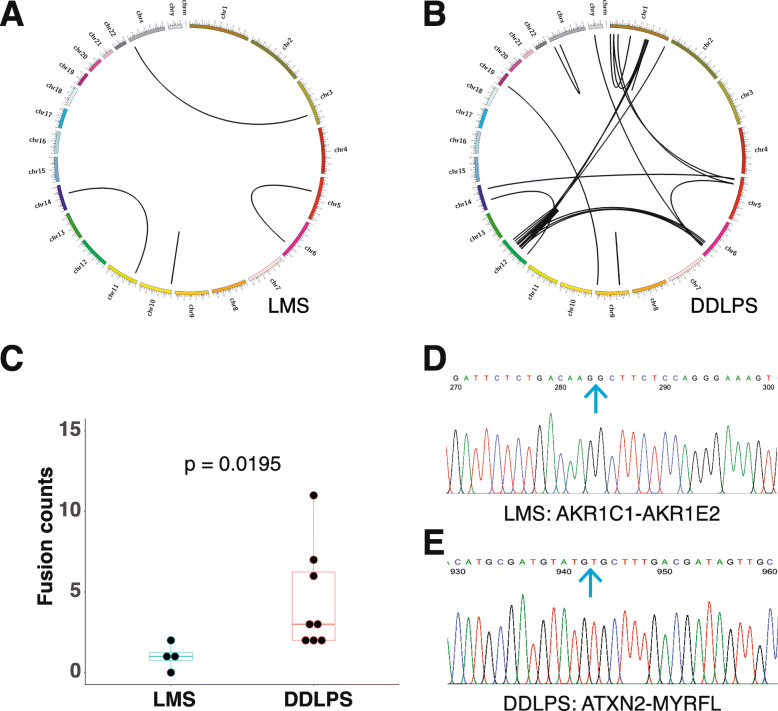
Distinct fusion patterns between DDLPS and LMS revealed by RNA-Seq. **a-b** Circos plot indicating genome-wide gene fusion events in LMS (a) and DDLPS (b). The lines link the partners of fusion. **c** Box plot of fusion events per sample in LMS and DDLPS. The median value is indicated by the line inside the box. The dots indicate the fusion counts in individual samples. **d-e** Electrophoresis traces of two example gene fusion events verified by RT-PCR in tandem with Sanger sequencing. The cerulean arrows indicate the position of fusion between the left- and right-side genes. *AKR1E1-AKR1E2* is detected in LMS (**d**) and *ATXN2-MYRFL* is detected in (**e**). The identities of all the RNA-seq predicted and Sanger sequencing verified fusion events are listed in Supplementary Table S1

**Fig. 7:**
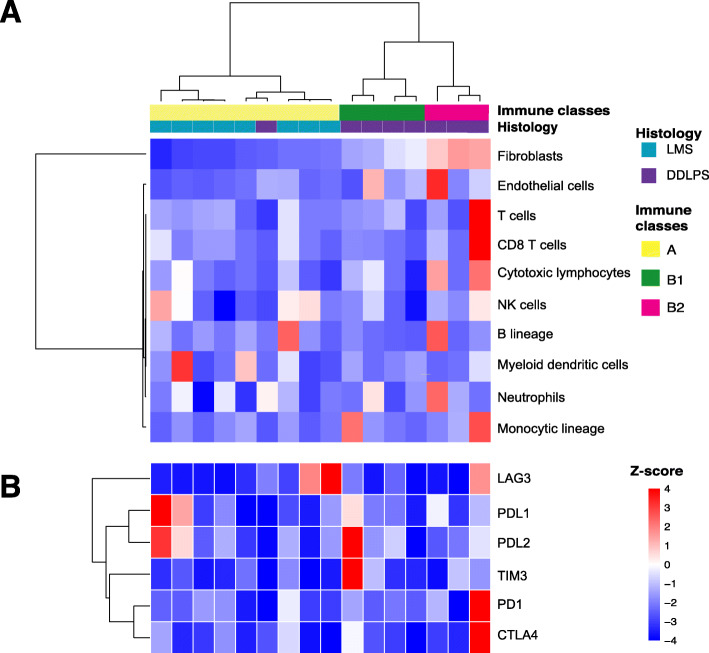
Stromal and immune cell infiltration in tumor microenvironment. **a** Unsupervised clustering of LMS (cerulean) and DDLPS (purple) samples by MCP-counter Z-scores. **b** Expression of genes related to immune checkpoints. Each column represents one patient and is aligned between (**a)** and (**b)**

In the RNA-seq analysis, we identified in 3 (out of 8) DDLPS patients 4 potential gene fusion transcripts and in all 8 LMS patients 36 fusion transcripts in total (Fig. 6 a-c and Supplementary Table S1). The distribution of fusion events is biased towards DDLPS over LMS (*p* = 0.0195, Fig. 6c). There is no recurrent fusion transcript identified probably due to the small sample size. Fusion transcripts involving chromosome 12 are only found in DDLPS, including both inter- and intra-chromosomal rearrangements (Fig. 6b). *MDM2* and *RAB3IP* are the most common fusion partners, and both are located in chromosome 12. There is also significant correlation between *MDM2/CDK4* amplification and chromosome 12 rearrangement (*P* < 0.001, Fig. 7b). Gene fusions can occur as a result of chromosomal rearrangements such as translocation, interstitial deletion, or inversion during DNA replication, which are more common in DDLPS than in LMS (Fig. 6 a-b). Therefore, it is not a surprise to see gene fusions more frequently in DDPLS, especially in chromosome 12 where ring or giant marker chromosomes often occur [4, 40]. Peptides generated from the identified fusion transcripts may be a potential source of tumor neoantigens that can be targeted to produce safer and patient specific CAR-T cells for immunotherapy [41]. Although RNA-seq by NGS method is good at high throughput survey of all possible gene fusion events in the sample, it relies on bioinformatic models to predict the fusion. This may potentially introduce errors. We selected all 4 predicted gene fusion transcripts in LMS and 8 out of 36 in DDLPS to verify with RT-PCR in tandem with Sanger sequencing. We were able to verify only 1 event in LMS but 7 out of 8 events in DDLPS (Fig. 6 d and Supplementary Table S1). This confirmed that gene fusions are relatively more frequent in DDLPS but scarce in LMS and further highlighted that neoantigen based immune therapy may have a higher success rate in DDLPS than in LMS.

### LMS and DDLPS have different profiles of tumor infiltrating immune cells

The status of tumor microenvironment (TME) is an important factor when immune therapy is considered. Previous studies revealed that STS immune subtypes are associated with response rate to PD1 blockade. To access the TME in LMS and DDLPS and compare the difference between them, we used MCP-counter to profile tumor infiltrating immune cells in the two subtypes following a recent study in STS (Fig. 7) [24]. Among the eight immune populations (T cells, CD8+ T cells, cytotoxic lymphocytes, natural killer cells, B cell lineage, monocytic lineage, myeloid dendritic cells and neutrophils) and two stromal populations (endothelial cells and fibroblasts) used by MCP-counter to categorize sarcomas immune classes (SIC), DDLPS has a higher signature score than LMS in both the non-immune cell populations (*p* = 0.002 for fibroblasts and *p* = 0.047 for endothelial cells). Increased endothelial cell signature score has been shown to associate with a high density of CD34+ endothelial cells and enhancer free endothelial-driven angiogenesis in STS [4]. The high fibroblasts signature score of DDLPS is consistent with its mesenchymal origin. The previous TCGA study suggested that CD8+ T cells are higher in DDLPS than in LMS (*P* < 0.01), but no such significance was observed in the current study (*P* = 0.76) [4].

Unsupervised clustering of the 16 DDLPS/LMS RNA samples according to their MCP-counter Z-scores revealed a bipartite pattern (Fig. 7a). All the LMS samples were classified to SIC A as defined by Petitprez et al., while most DDLPS samples except one were classified to SIC B [24]. The SIC B cluster can be further divided to two subclasses (B1 and B2) with the B2 subclass has a higher immune cell content in general. We also looked the expression of individual immune checkpoint molecules in the sample. It showed a higher representation of *PD-L1* in LMS and higher *TIM3*, *PD1* and *CTLA4* in DDLPS in some individual samples. Overall, the immune checkpoint point molecules are not highly active consistent to the observed ICI treatment efficiency in STS. However, the expression profiles of individual patients are worth to check when an ICI prescription is considered.

## Discussion

Our analysis revealed distinct mutation patterns and tumor microenvironments between LMS and DDLPS. This further testifies the diversity of STS and highlights the necessity of differential diagnosis and treatment of STS.

Genetic changes drive phenotypic change and eventually clinical manifestation and outcomes of disease. Both LMS and DDLPS belong to complex-karyotype STSs with an unbalanced karyotype and severe genomic aberrations. In this study, we revealed multiple chromosomal rearrangements in DDLPS, particularly the ones involving Chr12. These rearrangements were correlated with abundant gene amplification and fusion events as well. In contrary, LMS has fewer chromosomal level rearrangements, gene amplification and gene fusions (Figs. 3, 5, and 6). Notably high gene copy amplification and expression of *MDM2* and *CDK4* are detected. The two genes have been reported to promote the transformation of mesenchymal cells to DDLPS and it was suggested that they work together in the process [30, 35–37]. A simultaneous detection of highly upregulated gene expression upregulation and gene copy number of both genes in this report further strengths their role in DDLPS tumorigenesis. Therapies targeting to MDM2/CDK4 axis is expected to relief patients from DDLPS.

A higher degree of genetic scrambling as seen in the DDLPS predicts a better response to immune therapies, for example, the checkpoint inhibition. This notion is supported by several Phase II studies of immune checkpoint inhibition (ICI) therapies in sarcoma, including SARC028, Alliance A091401 and PEMBROSARC [42–44]. Results from these studies revealed that although LMS has a higher TMB than DDLPS, the response rate of ICIs in LMS is lower than in DDLPS. For both LMS and DDLPS, the response rate of ICIs in an unselected population is low, and TME immunological landscape profiling can assist in identification of patients who are likely to respond to immunotherapies. Using MCP-counter and RNA-seq results, we simultaneously quantified multiple cell populations and focused on the immune cells. Results from unsupervised clustering of MCP-counter Z-scores partitioned LMS and DDLPS in two distinct immune classes. All LMS coalesced to class A which is low of immune cell infiltration, fibroblasts and endothelial cells. The majority of DDLPS samples clustered to class B which has higher fibroblasts, endothelial cells and immune cell infiltration. Tumors in class A are generally considered “cold” with a low response rate to ICIs [45, 46]. For tumors in class B, two subgroups can be seen based on immune cell infiltrations. Class B1 has a relatively higher fibroblast and endothelial cells, and lower immune cell infiltration than class B2. In colon cancer, researchers have reported that immune and stromal classification was associated with molecular subtypes and patient’s prognosis [47]. Although we can correlate the histology, mutational profile and immune classification of LMS and DDLPS with the good or poor patient prognosis, dedicated prospective trials evaluating chemotherapy, targeted therapy, or immunotherapy would elucidate better the role of immunological landscape profiling in STS treatment. The integrated WES and RNA-seq analysis derived from the current study can potentially contribute to developing new biomarkers for patient screening and prognosis prediction.

At the molecular level, it is notable that prevailing cancer related mutations, including *TP53*, *AHNAK*, and *ATRX*, are exclusively in LMS and absent in DDLPS. Conversely, *HERC2* mutation only appears in DDLPS. These observations argue for that the two STS subtypes are derived from distinct cell origins and progressed through different mutational pathways. Diagnosis assisted by molecular profiling would bring additional value to patients besides pathological and histological approaches, especially when targeted or immune therapies are considered.

In spite of the above findings, several limitations remain in this study. Firstly, a relatively small number of patients with each histologic subtype were examined. This has prevented us to draw a clear-cut conclusion except for the few high frequency events we discussed above. STS is a rare disease, and it is even rarer for the individual subtypes. Therefore, analysis of few cases is still meaningful and has great reference value before a large number of cases can be available. Secondly, the evaluation of tumor microenvironment was based on RNA-seq of bulk cells. Although newer technologies such as single cell transcriptome analysis is available, due to the retrospective nature of this study, we could not obtain tissue specifically preserved and qualified for the single cell analysis. It also made it unlikely to verify the correlation of the molecular properties of the tumor with treatment in clinics. At last, we could not conduct survival analysis in the current cohort due to the relatively short follow-up time of the patients. These issues will be addressed in future studies and the insights we gained in the current study will help us to make appropriate patient stratification.

## Conclusion

In conclusion LMS and DDPLS are distinct diseases as supported by our analysis of mutation pattern, gross genomic stability, and tumor infiltrating immune cell profiles. Chromosomal rearrangement may result in gene amplification and fusion, which are displayed the most obviously in Chr12 in DDLPS. These observations are consistent to the altered gene expression patterns in the two types of sarcoma. Neoantigens produced by gene fusion may open up new avenues for personalized immunotherapy in STS.

## Supplementary information

**Additional file 1: Supplementary Table S1.** list of gene fusion events and their sequences, and the oligos used and products detected by RT-PCR.

**Additional file 2: Supplementary Table S2.** SNV list

**Additional file 3: Supplementary Table S3.** LMS_amplified_gene list in 0.95 confidence level

**Additional file 4: Supplementary Table S4.** LMS_deleted_gene list in 0.95 confidence level

**Additional file 5:. Supplementary Table S5.** DDLPS_ amplified_gene list in 0.95 confidence level

**Additional file 6: Supplementary Table S6.** RNA expression from RNA-seq analysis

**Additional file 7: Supplementary Table S7.** Pathways enriched from RNA-seq analysis

## Data Availability

The data analyzed in this article is available from the corresponding author on reasonable request.

## Abbreviations

STS: Soft-tissue sarcoma
WES: Whole exome sequencing
LPS: Liposarcoma
DDLPS: De-differentiated LPS
LMS: Leiomyosarcoma
CAR-T: Chimeric Antigen Receptor T
SNV: Single nucleotide variations
SCNA: Somatic copy-number alteration
CNA: Copy-number alteration
GATK: Genome Analysis Toolkits
CIN: Chromosomal instability
MSI: Microsatellite Instability
TPM: Transcripts Per Million
KEGG: Encyclopedia of Genes and Genomes
GSEA: Gene Set Enrichment Analysis
TMB: Tumor mutation burden
ICI: Immune checkpoint inhibitor
